# Supplementary material for: HER2/CEP17 ratio is associated with pCR after HER2-directed neoadjuvant treatment in the phase III NeoALTTO trial
Source: Breast. 2025 Dec 22;85:104679. doi: 10.1016/j.breast.2025.104679 (PMC12813240; doi:10.1016/j.breast.2025.104679)
Supplement: Multimedia component 1 [file mmc1.doc]

Original Report

**HER2/CEP17 Ratio is associated with pCR after HER2-directed Neoadjuvant Treatment in the Phase III NeoALTTO Trial**

**Christian F Singer^1,2,3^, Franz Koenig^4^, Stephanie Kacerovsky-Strobl^3^, Sabine Danzinger^1^, Christine Brunner^5^, Christoph Suppan^6,3^, Christine Deutschmann^1^, Marija Balic^6,7,3^, Richard Greil^8,3^, Daniel Egle^5,3^, Evandro de Azambuja^9^,** **Serena Di Cosimo^10^, Anup Choudhury^11^, Michael Gnant^2,3^**

^1^Department of Obstetrics, Gynecology and Gynecological Oncology, Medical University of Vienna, Vienna, Austria; ^2^Comprehensive Cancer Center, Medical University of Vienna, Vienna, Austria; ^3^Austrian Breast and Colorectal Cancer Study Group, Vienna, Austria; ^4^Institute of Medical Statistics, Center for Medical Data Science, Medical University of Vienna, Vienna, Austria; ^5^Department of Obstetrics and Gynecology, Medical University of Innsbruck, Innsbruck, Austria; ^6^Division of Oncology, Department of Internal Medicine, Medical University of Graz, Graz, Austria; ^7^Division of Hematology/Oncology, Department of Medicine, University of Pittsburgh School of Medicine, Pittsburgh, PA, United States; ^8^Salzburg Cancer Research Institute-CCCIT, Cancer Cluster Salzburg, Paracelsus Medical University Salzburg, Salzburg, Austria; ^9^Department of Medical Oncology, Institute Jules Bordet, Université libre de Bruxelles (U.L.B) and Hôpital Universitaire de Bruxelles (H.U.B), Brussels, Belgium; ^10^Department of Advanced Diagnostics, Fondazione IRCCS Istituto Nazionale dei Tumori, Milan, Italy; ^11^ Novartis Healthcare Pvt. Ltd. Hyderabad, India

**Corresponding Author:**

Prof. Christian F Singer, MD, MPH
Department of Obstetrics, Gynecology and Gynecological Oncology

Medical University of Vienna
Waehringer Guertel 18-20, 1090 Vienna, Austria
Phone: [+43 1 40400 2801](tel:%2B43 1 40400 2801)0
Email: [christian.singer@meduniwien.ac.at](mailto:christian.singer@meduniwien.ac.at)

**Keywords:** Breast cancer; HER2-positive; neoadjuvant; HER2/CEP17 ratio; pCR

**HIGHLIGHTS**

- Efficacy of anti-HER2 therapy related to HER2 overexpression in HER2+ eBC is unknown.
- We evaluated HER2/CEP17 ratio, pCR, and EFS correlation in the NeoALTTO trial.
- We found that pre-treatment HER2/CEP17 ratio by FISH predicts pCR but not of EFS.
- HER2/CEP17 ratio is a readily available and simple predictor of pCR in HER2+ eBC without chemotherapy.

**ABSTRACT**

**Purpose:** In early breast cancer, HER2-directed therapies are approved for the treatment of patients with HER2-positive invasive breast cancer as defined by HER2 protein overexpression, or *HER2* gene amplification with *HER2/CEP17* ratios ≥ 2.2. Beyond this cut-off, however, it is unknown whether the efficacy of HER2-directed therapy improves with increasing *HER2/CEP17* ratios. We evaluated whether quantitative assessment of the *HER2/CEP17* ratio predicts pathological complete response (pCR) and event-free survival (EFS) in patients treated with neoadjuvant HER2-based regimen in the prospective phase III NeoALTTO trial.

**Patients and Methods:** 455 women with HER2-positive early breast cancer, who had received neoadjuvant trastuzumab and/or lapatinib, together with 12 cycles of weekly paclitaxel, were included in this analysis. The *HER2/CEP17* ratio in the primary tumor samples was correlated with pCR and survival outcome.

**Results:** The median *HER2/CEP17* ratio was 5.1 (range: 1.1 - 100.0), and ratios were not associated with age, hormone receptor (HR) status, or any other clinicopathological variable analyzed. The log *HER2/CEP17* ratio significantly predicted pCR in both univariate (OR: 1.83; 95% CI: 1.11 - 3.01, p=0.0176) and multivariate analysis (OR: 1.79; 95% CI: 1.07 - 2.99, p=0.0257). Higher *HER2/CEP17* ratios were, however, not associated with improved EFS (adjusted HR=0.795; p=0.3537). A pCR prediction model including *HER2/CEP17* ratio, treatment arm, and HR status improved the predictive strength of treatment arm alone from a ROC AUC value of 0.60 to 0.69.

**Conclusion:** In patients treated with HER2-based neoadjuvant therapy, quantitative analysis of the readily available pretreatment *HER2/CEP17* ratio by FISH is predictive of pCR**.**

**INTRODUCTION**

Human Epidermal Growth Factor Receptor 2 (HER2) is amplified/overexpressed in approximately 20% of human breast carcinomas and was identified as a therapeutic target several years ago.[[1]](#endnote-2) The subsequent development of the HER2-targeting antibody trastuzumab has dramatically improved the outcomes of HER2-overexpressing breast cancer patients previously considered at dismal prognosis.[[2]](#endnote-3),[[3]](#endnote-4) In addition, the HER2 tyrosine kinase inhibitor lapatinib has been demonstrated to improve time to progression (TTP) in patients progressing on trastuzumab, when combined with capecitabine.[[4]](#endnote-5),[[5]](#endnote-6) The NeoALTTO trial was conducted because trastuzumab and lapatinib have complementary mechanisms of action, and their combination exerts supra-additive anti-tumor activity in preclinical models. This study demonstrated a significantly higher pCR in patients receiving both lapatinib and trastuzumab, than in those receiving either trastuzumab or lapatinib alone.[[6]](#endnote-7) In the recently presented final analysis of NeoALTTO performed after a follow-up of 9.7 years, patients with pCR had a significantly higher 6-year EFS (77% vs 61%, HR 0.48, 95%, CI 0.31 - 0.73, p = 0.0008) and overall survival (OS) (88% vs 72%, HR 0.37, 95% CI 0.20 - 0.63, p = 0.0004) compared with those who did not.[[7]](#endnote-8)

We have previously measured the *HER2/chromosome enumeration probe (CEP17)* ratio in a cohort of 120 HER2-overexpressing patients who had not received (neo)adjuvant trastuzumab, but were treated with HER2-directed therapy in the metastatic setting^12^. In this population, a *HER2/CEP17* ratio >6 independently predicted for a shorter time to first metastasis, and was also associated with a higher response rate and an increased progression-free survival (PFS) to trastuzumab.[[8]](#endnote-9) More recently, we described in a subset of women participating in the prospective neoadjuvant ABCSG-24 and ABCSG-32 trials, that primary tumors with a *HER2/CEP17* ratio >6 were more likely to achieve pCR in response to trastuzumab. Interestingly, the association between high *HER2* amplification and pCR was almost exclusively confined to HR-positive cases[[9]](#endnote-10).

We have therefore now analyzed in the prospective, randomized phase III NeoALTTO trial whether the degree of *HER2* amplification, is associated with pCR and is predictive of long-term outcome in patients treated with HER2-directed therapy. This is particularly important since recent antibody-drug conjugate (ADC) developments such as trastuzumab-deruxtecan (T-DXd) are also effective in low HER2 expressing advanced breast cancer, and may work independently of the magnitude and possible intra-tumoral heterogeneity of HER2 expression.[[10]](#endnote-11),[[11]](#endnote-12),[[12]](#endnote-13),[[13]](#endnote-14) While data from the neoadjuvant setting are still lacking, it has been hypothesized that T-DXd is also effective in HER2-expressing breast cancer irrespective of the *HER2/CEP17* ratio.

**RESULTS**

**Patient Characteristics**

Of the 455 women who had been randomized in NeoALTTO (BIG 01–06), a *HER2/CEP17* ratio could be calculated in a subset of 281 patients (“FISH cohort”) (Figure 1). This subset was representative of the overall study population (not shown). Of these, 94 (33.5%) achieved pCR, and in this group, the median *HER2/CEP17* ratio was 5.55 (range 1.10 - 40.00). The median intratumoral *HER2/CEP17* ratio in patients who had not experienced pCR was 5.00 (range 1.45 - 100.00) (Table 1). Patient demographics and tumor characteristics in the FISH cohort and according to the achievement of pCR are shown in Table 1. In brief, patients included in this analysis had a median age of 50 years (range 23 - 80 years) and a median primary tumor size of 4 cm (range 2.1 - 9.5 cm) at baseline; 51% had HR-positive disease and 73% of all patients had involved axillary lymph nodes at baseline. Intermediate (38%) and high (45%) grade tumors were present in the majority of cases. Invasive ductal histology predominanted in 86% of cases, while lobular histology was present in 5% of cases.

**Correlation between *HER2/CEP17* ratio and clinicopathological variables**

The *HER2/CEP17* ratio did not show a statistically significant correlation with the metrical variables age, tumor size, HR status, histologic grade, histologic subtype, or menopausal status (Table 2), it did, however, show a weak correlation with the percentage of invasive tumor cells with complete membrane staining (i.e. 3+; Pearson´s correlation coefficient r=0.26, p=0.0036) (Supplementary figure 1)

***HER2/CEP17* ratio and prediction of pCR**

Correlations between the log *HER2/CEP17* ratio in individual patients and pCR in the overall population, in the respective treatment subgroups, and in HR+ and HR- patients are shown in Figure 2 A-F. To investigate the effect of the *HER2/CEP17* ratio on pCR, we first used a simple logistic regression model, in which the effects of patient age, menopausal status, treatment arm, *HER2/CEP17* ratio, and other tumor-biological variables were evaluated with respect to the dependent outcome variable pCR. In univariate analysis, we found that the log *HER2/CEP17* ratio had a statistically significant association with pCR (OR: 1.83; 95% CI: 1.11 - 3.017, p=0.0176, Table 2). The absence of hormone receptors was also associated with a higher probability of achieving pCR (OR: 2.149; 95% CI: 1.451 - 3.184, p=0.0001). Treatment allocation to lapatinib + trastuzumab vs. trastuzumab alone was also predictive of pCR (OR: 2.515; 95% CI: 1.565 - 4.042; p<0.0001, Table 2), while lapatinib monotherapy did not improve pCR compared to trastzumab (OR: 0.782; 95% CI: 0.470 - 1.299). To further explore the association with the *HER2/CEP17* ratio, we also analyzed the ratio as a categorical variable after subdividing the available *HER2/CEP17* ratios into quartiles (quartile 1: *HER2/CEP17* ratio ≤3.92; quartile 2: *HER2/CEP17* ratio: 3.93 - ≤5.14; quartile 3 *HER2/CEP17* ratio 5.15 - ≤7.15; quartile 4: *HER2/CEP17* ratio >7.15), and found that in the univariate model, tumors which exhibited a *HER2/CEP17* ratio in the lowest quartile, had a significantly lower chance of achieving pCR than tumors whose ratio was in any of the higher quartiles (p=0.048).

In the multiple regression model, however, only the log *HER2/CEP17* ratio was used due to the skewed distribution of *HER2/CEP17* ratios in the overall population (Figure 3A). Indeed, when a stepwise logistic regression model was performed, which adjusted for the covariates HR and treatment arm, the log *HER2/CEP17* ratio remained a statistically significant predictor of pCR (OR: 1.793; 95% CI: 1.073 - 2.996, p=0.0257, Table 2). We also show the distribution of HER2/CEP17 ratios in the subgroup of patients treated with trastuzumab alone (Figure 3B), trastuzumab and lapatinib (Figure 3C), lapatinib alone (Figure 3D), and in HR+ (Figure E) and in HR- patients (Figure F) and found similar distributions, although the respective numbers were small.

We then analyzed whether the addition of the HR status and the *HER2/CEP17* ratio could improve the predictive strength of the treatment modality. While the ROC for treatment arm (trastuzumab vs. lapatinib vs. lapatinib + trastuzumab) had an AUC of 0.62 [95% CI: 0.55 - 0.69], the AUC for HR status was 0.58 [95% CI: 0.52 - 0.64], and 0.60 [95% CI 0.53 - 0.67], for the *HER2/CEP17* ratio. By combining all three variables in a single model, the AUC could be increased to 0.69 [95%-CI: 0.62 - 0.76] (Figure 4).

***HER2/CEP17*** **ratio and long-term outcomes**

At a median follow-up of 6.7 years [IQR: 5.7 - 6.8], 73 of 281 (26%) patients, in whom *HER2/CEP17* ratios were evaluable, had experienced a disease event and 45 of 281 (16%) had died. The independent effect of the log *HER2/CEP17* ratio on EFS and OS was assessed using Cox proportional hazard models adjusted for age, tumor size, nodal status, tumor grade, as well as ER and PR expression. In multivariate analyses, an increased *HER2/CEP17* ratio was neither associated with a significantly prolonged EFS (adjusted HR=0.795, p=0.35 Cox regression analysis) nor improved OS (adjusted HR=0.792, p=0.44).

We then grouped the available *HER2/CEP17* ratios in the overall study population into quartiles and assessed EFS and OS for each of the respective quartiles after a median follow-up of 6.7 years [IQR: 5.7 - 6.8]. The corresponding Kaplan-Maier curves for each of the quartiles in terms of EFS and OS are shown in Figure 5A and 5B, and did not reveal significant differences (logrank test p=0.3537 for EFS, and p=0.5596 for OS). Kaplan-Maier curves for EFS and OS according to the *HER2/CEP17* ratio for each of the quartiles in HR negative and HR positive tumors and for each of the treatment arms are shown in Supplementary Figures 2A-F (for EFS) and 3A-F (for OS).

**DISCUSSION**

To date the identification of early breast cancers that are likely to respond to HER2-targeted treatment is based on semiquantitative IHC protein assessment and dichotomized in situ hybridization.[[14]](#endnote-15) Although both tests determine whether a tumor is a potential target for HER2-targeted therapy, almost 23% of women with early breast cancer treated with trastuzumab-based chemotherapy relapse within the first 10 years of diagnosis.[[15]](#endnote-16) Because of this relatively poor outcome prediction, alternative methods have been investigated to improve the predictive accuracy of conventional HER2 testing, but to date no established biomarker exists.

Scaltriti et al. measured HER2 protein levels by a proximity-based immunoassay that quantitatively measures HER2 total protein and correlated with IHC HER2 testing. Using this assay, the authors found a positive correlation between the truncated form of the HER2 receptor p95HER2 and total HER2 levels in the 60% of cases. High levels of p95HER2 were predictive of pCR, particularly in HR-positive cases. High HER2 expression was similarly correlated with an increased pCR rate, although the association was independent of the HR status.

In an exploratory biomarker analysis from the phase III KATHERINE study of adjuvant T-DM1 recently investigated the relationship between invasive disease-free survival (IDFS) and HER2 gene expression. The authors found that high versus low HER2 gene expression in residual disease was associated with worse outcomes with trastuzumab [HR, 2.02; 95% confidence interval (CI), 1.32-3.11], but IDFS with T-DM1 was independent of HER2 expression level (HR, 1.01; 95% CI, 0.56-1.83).[[16]](#endnote-17) Similarly, Mogica et al., found that a reduced HER2 expression by ICH following neoadjuvant trastuzumab/pertuzumab-containing chemotherapy was associated with lower recurrence rates in HER2 ICH 3+ breast cancer.[[17]](#endnote-18)

Our finding of an improved predictive strength of the *HER2/CEP17* ratio by adding simple clinical parameters such as HR status and treatment modality to the model again demonstrates that using routine clinical parameters is clinically meaningful. An integrated analysis of mRNA-seq and DNA exome sequencing data from CALGB 40601 used an Elastic Net regularized regression approach, utilizing only gene signatures, which predicted pCR in the validation sets with an AUC of 0.76. They concluded that models containing gene signatures, clinical features, and DNA information were better pCR predictors than models containing a single data type.[[18]](#endnote-19),[[19]](#endnote-20)

In the same population in which we performed our analysis on the *HER2/CEP17* ratio, Nuciforo et al. found that patients who exhibited pCR after neoadjuvant anti-HER2-based treatment had significantly better EFS and OS for patients achieving pCR after neoadjuvant anti-HER2-based therapies.^7^ The probability of achieving pCR was particularly high for patients with HR-negative tumors compared to those with triple-positive disease. When pCR was achieved in the HR-negative cohort, the 6-year EFS increased from 57% to 77% when pCR was observed compared to HR-negative patients without pCR. In a protocol-defined sub-study Fumagalli et al. used NeoALTTO tumor samples in order to evaluate potential associations between intratumoral HER2 gene expression levels and PAM50 subtypes, and the outcome parameters pCR and EFS. They identified pretreatment HER2 gene expression levels as the most significant predictors of pCR, followed by the HER2-enriched tumor subtype. Interestingly, most of the observed pCRs clustered in the group of patients with high HER2 and low ESR1 expression levels, while those in the low HER2 group rarely achieved a pCR. In analogy to our results, they also did not find a correlation with EFS, and the authors suggest that the analysis was underpowered.^23^

The fact that the treatment strategies in NeoALTTO included both extracellular (trastuzumab) and intracellular (lapatinib) inhibitory strategies, which were given individually or concomitantly, provided an ideal setting to investigate potential *HER2/CEP17* ratio-associated differences in efficacy. Nevertheless, our study is somewhat limited by the small sample size in individual treatment arms, and by the inclusion of tumors with a *HER2/CEP17* ratio of >2.2 (as per the guidelines at the time of study recruitment)[[20]](#endnote-21).

The relatively small number of patients (n=281) in our study with an available *HER2/CEP17* ratio (n=281) is therefore also the most likely explanation between the lack of association between pCR and long-term outcome. Based on a pooled analysis from 12 international trials with almost 12.000 patients, pCR is now an established surrogate endpoint for prediction of long-term clinical benefit. This is particularly true for HER2-positive, HR-negative tumours who received trastuzumab (With a HR for EFS of 0.15 (95 CI: 0.09-0.27) and a HR for OS of 0.08, (95% CI: 0.03-0.22).[[21]](#endnote-22) It cannot be excluded that, with larger patient numbers, such a correlation would also emerge in the NeoALTTO study population. Also, adjuvant systemic treatment may have impacted on outcomes and thereby compromised any potential long-term survival difference. The validity of neoadjuvant trials in predicting the outcome of large adjuvant clinical studies has been elegantly addressed in a publication by DeMichele et al., who, by using the FDA meta-analysis could demonstrate that there was no discordance at between the observed pCR difference in NeoALTTO and the observed HR in ALTTO, and who emphasized the importance of appropriately modelling the two endpoints when designing clinical trials.^24^

In summary, we found that increasing *HER2/CEP17* ratios were associated with a significantly higher probability of achieving pCR in both uni- and multivariable analyses. We also developed a pCR prediction model that included treatment arm and HR status, and allowed us to improve the discriminatory capability to a final AUC value of 0.69. Our findings are particularly important since, in contrast to the advanced setting where the mere presence of HER2 protein by IHC predicts response to T-DXd, the efficacy of currently available (neo)adjuvant regimens is not only restricted to HER2 overexpression/amplification but also significantly depends on the degree of HER2 amplification.

**PATIENTS AND METHODS**

**Study design**

The NeoALTTO trial (BIG 01–06) is a randomized, multicenter, open-label, phase III study that recruited 455 patients with early HER2-positive breast cancer between 2008 and 2010. The design of NeoALTTO has been reported in detail previously.^6^ In brief, patients were randomized 1:1:1 to receive either lapatinib, trastuzumab, or a combination of both for 6 weeks, followed by additional 12 weeks of the assigned anti-HER2 therapy in combination with weekly paclitaxel. Patients underwent surgery within 4 weeks after the last dose of paclitaxel, and received 3 cycles of adjuvant FEC q3w and their assigned anti-HER2 therapy for 34 weeks.[[22]](#endnote-23)

The primary objective was the rate of pCR, defined as the absence of residual invasive cancer in surgical breast specimens (ypT0/is). Secondary endpoints included EFS, defined as breast cancer relapse, second primary malignancy or death without recurrence; and OS.

**Statistical analysis**

For categorical variables, absolute numbers and percentages, for metric variables, mean, standard deviation, median, minimum and maximum are reported. Associations between the *HER2/CEP17* ratio and metric clinicopathological variables such as age and primary tumor size were evaluated by Spearman correlation coefficients. To identify potential differences in the *HER2/CEP17* ratio with respect to categorical variables such as menopausal and HR status, the non-parametric Mann-Whitney-U and Kruskal-Wallis tests were performed.

The association between the *HER2/CEP17* ratio and pCR was evaluated using a logistic regression model. Covariates were adjusted by stepwise logistic regression. Due to the skewness of the *HER2/CEP17* ratio data, the log-transformed *HER2/CEP17* ratio was used in all analyses.

To evaluate the association with time-to-event outcomes such as EFS and OS, we performed log-rank tests and Cox regression models. Time-to-event data were visualized using Kaplan-Meier plots.

**CONFLICT OF INTEREST STATEMENT**

**Christian F. Singer** reports disclosures related to paid consultancies (AstraZeneca, Novartis, Gilead) and research grants (Novartis, Amgen, Gilead, Daiichi Sankyo, AstraZeneca); **Franz Koenig** reports to have no disclosures; **Stephanie** **Kacerovsky-Strobl** reports to have no disclosures; **Sabine Danzinger** reports disclosures related to honoraria (AstraZeneca, Eli Lilly, Roche), consulting/advisory role (Roche) and travel/accommodations/expenses (Amgen, Daiichi Sankyo, Eli Lilly, MSD, Novartis, Pfizer, Roche, Sandoz); **Christine Brunner** reports disclosures related to honoraria (Amgen, Saegen, Daiichi Sankyo/AstraZeneca, Gilead), consulting/advisory role (Gilead, Novartis) and travel/accommodations/expenses (Daiichi Sankyo/AstraZeneca); **Christoph** **Suppan** reports disclosures related to paid consultancies (Novartis, Eli Lilly, Pfizer, Daiichi Sankyo, AstraZeneca, Pierre Fabre) and honoraria (Novartis, Eli Lilly, Pfizer, Daiichi Sankyo, AstraZeneca, Pierre Fabre); **Christine Deutschmann** reports disclosures related to honoraria (Stemline, AstraZeneca, Lilly, Gilead), research funding (Novartis); consulting/advisory role (Novartis, Stemline) and travel/20egression20on/expenses (AstraZeneca, Roche, Novartis, Daiichi Sankyo, Pfizer, Stemline); **Marija Balic** reports disclosures related to honoraria (Amgen, AstraZeneca, Celgene, Daiichi Sankyo, Eli Lilly, MSD, Novartis, Pierre Fabre, Pfizer, Roche, Samsung, Gilead), paid consultancies (Amgen, AstraZeneca,  Celgene, Daiichi Sankyo, Eli Lilly, MSD, Novartis, Pierre Fabre, Pfizer, Roche, Samsung, Gilead), speakers’ bureau (Amgen, AstraZeneca, Celgene, Daiichi Sankyo, Eli Lilly, MSD, Novartis, Pierre Fabre, Pfizer, Roche, Seagen, Gilead), research grants/other funding (Pfizer, Eli Lilly, AstraZeneca, Daiichi Sankyo, Pierre Fabre) and travel/accommodations/expenses (Eli Lilly, MSD, Novartis, Pierre Fabre, Pfizer, Roche, Gilead); **Richard** **Greil** reports disclosures related to paid consultancies (Celgene, Novartis, Roche, BMS, Takeda, Abbvie, AstraZeneca, Janssen, MSD, Merck, Gilead, Daiichi Sankyo, Sanofi), honoraria (Celgene, Roche, Merck, Takeda, AstraZeneca, Novartis, Amgen, BMS, MSD, Sandoz, Abbvie, Gilead, Daiichi Sankyo, Sanofi), participation on a Data Safety Monitoring Board or Advisory Board (Celgene, Novartis, Roche, BMS, Takeda, Abbvie, AstraZeneca, Janssen, MSD, Merck, Gilead, Daiichi Sankyo, Sanofi), stock ownership (Novo Dordisk, Lilly) and other funding (Celgene, Roche, Merck, Takeda, AstraZeneca, Novartis, Amgen, BMS, MSD, Sandoz, Abbvie, Gilead, Daiichi Sankyo);  **Daniel Egle** reports disclosures related to honoraria/travel grants/paid consultancies (Amgen, AstraZeneca, Daiichi Sankyo, Gilead, Lilly, MSD, Novartis, Pfizer, Pierre Fabre, Roche, Sandoz, Seagen); **Evandro de Azambuja** reports financial disclosures related to honoraria and/or advisory board (Roche/GNE, Novartis, Seagen, Zodiac, Libbs, Pierre Fabre, Lilly, AstraZeneca, MSD, Gilead Sciences), travel grants (AstraZeneca, Gilead), research grants to his institution (Roche/GNE, AstraZeneca, GSK/Novartis, Gilead Sciences, Seagen/Pfizer) as well as non-financial disclosures (ESMO director of Membership 2023-2025, BSMO President 2023-2026); **Serena Di Cosimo** reports disclosures related to consulting/advisory role (Pierre-Fabre, MEDSIR), speakers’ bureau (AstraZeneca) and research funding (Fondazione AIRC); **Anup Choudhury** reports disclosures related to his employment at Novartis; **Michael** **Gnant** reports disclosures related to personal fees/travel support (Amgen, AstraZeneca, Bayer, Daiichi Sankyo, Eli Lilly, EPG Health (IQVIA), Menarini-Stemline, MSD, Novartis, Pierre Fabre, Veracyte).

**DATA STATEMENT**

The data generated in this study are not publicly available but are available upon reasonable request from the corresponding author.

**TABLES**

Table 1: Patient demographics

|  | *PCR=No* | *PCR=Yes* | *All* |
| --- | --- | --- | --- |
| **HER2/CEP17 Ratio** | | | |
| Mean (Std) | 6.18 (7.52) | 6.93 (4.73) | 6.42 (6.71) |
| Median (Min-Max) | 5.00 (1.45-100.00) | 5.55 (1.10-40.00) | 5.14 (1.10-100) |
| n | 187 | 94 | 281 |
| **Log HER2/CEP17 Ratio** | | | |
| Mean (Std) | 1.6 (0.5) | 1.8 (0.5) | 1.7 (0.5) |
| Median (Min-Max) | 1.6 (0.4-4.6) | 1.7 (0.1-3.7) | 1.6 (0.1-4.6) |
| n | 187 | 94 | 281 |
| **Age (years) at baseline** | | | |
| Mean (Std) | 50.1 (11.45) | 49.4 (10.79) | 49.9 (11.22) |
| Median (Min-Max) | 50 (23-79) | 49 (23-80) | 50 (23-80) |
| **Tumor size (cm) at baseline** | | | |
| Mean | 9.491 | 11.306 | 10.129 |
| Std | 13.2215 | 16.1067 | 14.3112 |
| Median | 4.100 | 4.000 | 4.000 |
| Min | 2.06 | 2.10 | 2.06 |
| Max | 95.00 | 90.00 | 95.00 |
| **Randomised treatment arm** | | | |
| Lapatinib | 116/295 (39%) | 38/160 (24%) | 154/455 (34%) |
| Lapatinib + trastuzumab | 74/295 (25%) | 78/160 (49%) | 152/455 (33%) |
| Trastuzumab | 105/295 (36%) | 44/160 (28%) | 149/455 (33%) |
| **Hormone receptor status** | | | |
| Negative | 125/295 (42%) | 98/160 (61%) | 223/455 (49%) |
| Positive | 170/295 (58%) | 62/160 (39%) | 232/455 (51%) |
| **Clinical N stage at baseline** | | | |
| N0 | 72/295 (24%) | 51/160 (32%) | 123/455 (27%) |
| N1 | 180/295 (61%) | 80/160 (50%) | 260/455 (57%) |
| N2 | 28/295 (9%) | 16/160 (10%) | 44/455 (10%) |
| N3 | 12/295 (4%) | 10/160 (6%) | 22/455 (5%) |
| NX | 3/295 (1%) | 3/160 (2%) | 6/455 (1%) |
| **Histologic grade** | | | |
| G1 | 9/295 (3%) | 3/160 (2%) | 12/455 (3%) |
| G2 | 120/295 (41%) | 52/160 (33%) | 172/455 (38%) |
| G3 | 125/295 (42%) | 80/160 (50%) | 205/455 (45%) |
| GX | 41/295 (14%) | 25/160 (16%) | 66/455 (14%) |
| **Invasive histologic type** | | | |
| Ductal carcinoma | 247/295 (84%) | 144/160 (90%) | 391/455 (86%) |
| Lobular carcinoma | 16/295 (5%) | 5/160 (3%) | 21/455 (5%) |
| Others or missing | 32/295 (11%) | 11/160 (7%) | 43/455 (9%) |
|  |  |  |  |
| n represents number of patients contributing to summary statisics. Percentages are based on n (number of valid values). Percentages not calculated if n < 10 | | | |

Table 2: Simple and stepwise logistic regression models with pCR as dependent outcome variable

| **Model** | **Positive/total (%)** | **Simple logistic** |  | **Stepwise logistic** |  |
| --- | --- | --- | --- | --- | --- |
|  |  | **Odds ratio** | **P value** | **Odds ratio** | **P -value** |
| **log HER2/CEP17 ratio** |  | 1.831 (1.111;3.017) | 0.0176 | 1.793 (1.073; 2.996) | 0.0257 |
|  |  |  |  |  |  |
| **HER2/CEP17 ratio (Quartils)** |  |  | 0.0482 |  | ***** |
| 1.Q: R<=3.92 | 14/71 (19.7%) | 1 |  |  |  |
| 2.Q: 3.93<R<=5.14 | 26/70 (37.1%) | 2.406 (1.126; 5.142) |  |  |  |
| 3.Q: 5.15 <R<=7.15 | 26/70 (37.1%) | 2.406 (1.126; 5.142) |  |  |  |
| 4.Q: R>7.15 | 28/70 (40%) | 2.714 (1.275; 5.777) |  |  |  |
|  |  |  |  |  |  |
| **Treatment Arm** |  |  | <.0001 |  | 0.0005 |
| Trastuzumab | 44/149 (29.5%) | 1 |  |  |  |
| Lapatinib | 38/154 (24.7%) | 0.782 (0.470; 1.299) |  | 0.778 (0.400; 1.512) |  |
| Trastuzumab + Lapatinib | 78 /152 (51.3%) | 2.515 (1.565; 4.042) |  | 2.566 (1.363; 4.831) |  |
|  |  |  |  |  |  |
| **Tumor Size** |  | 1.009 (0.995; 1.022) | 0.1992 |  | NS |
|  |  |  |  |  |  |
| **HR** |  |  | 0.0001 |  | 0.0054 |
| HR positive | 62/232 (26.7%) | 1 |  | 1 |  |
| HR negative | 98/223 (44%) | 2.149 (1.451; 3.184) |  | 2.133 (1.251; 3.636) |  |
|  |  |  |  |  |  |
| **Age** |  | .994 (0.977; 1.012) | 0.5135 |  |  |
|  |  |  |  |  |  |
| **Menopause** |  |  | 0.5471 |  |  |
| Premenopausal | 85/233 (36.5%) | 1 |  |  |  |
| Postmenopausal | 75/222 (33.8%) | 0.888 (0.604; 1.306) |  |  |  |
|  |  |  |  |  |  |
| **Nodal status** |  |  | 0.0765 |  | NS |
| NO | 51/123 (41.5%) | 1 |  |  |  |
| N1 | 80/260 (30.8%) | 0.627 (0.402; 0.979) |  |  |  |
| N2, N3 or NX | 29/72 (40.3%) | 0.952 (0.527; 1.721) |  |  |  |
|  |  |  |  |  |  |
| **Grade** |  |  | 0.0528 |  | NS |
| G1, G2 | 55/184 (29.9%) | 1 |  |  |  |
| G3, GX | 105/271 (38.8%) | 1.484 (0.995; 2.211) |  |  |  |
|  |  |  |  |  |  |
| **Histology** |  |  | 0.1897 |  | NS |
| Ductal carcinoma | 144/391 (36.8%) | 1 |  |  |  |
| Lobular carcinoma | 5/21 (23.8%) | 0.536 (0.192; 1.495) |  |  |  |
| Others or missing | 11/43 (25.6%) | 0.590 (0.288; 1.206) |  |  |  |

**FIGURES**

**Figure 1:** CONSORT diagram of patient selection for secondary analysis in the


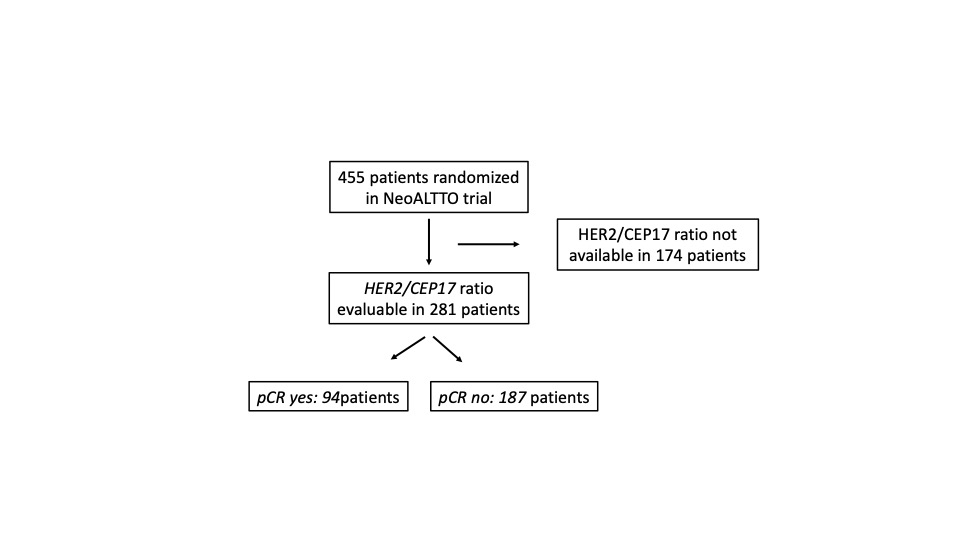
NeoALTTO trial. Starting with 455 patients, 174 samples were removed due to non existant / incomplete HER2/CEP17 ratios, thus leaving 281 patients. Of these, 94 had a complete pathological remission (pCR), whereas 187 did not.

**Figure 2:** Waterfall plot depicting A: overall pCR (blue) and non-pCR (red) according to the log HER2/CEP17 ratio in individual patients; B: pCR (blue) and non-pCR (red) according to the log HER2/CEP17 ratio in trastuzumab-treated patients; C: pCR (blue) and non-pCR (red) according to the log HER2/CEP17 ratio in trastuzumab and lapatinib-treated patients; D: pCR (blue) and non-pCR (red) according to the log HER2/CEP17 ratio in lapatinib-treated patients; E: pCR (blue) and non-pCR (red) according to the log HER2/CEP17 ratio in HR+ patients; F: pCR (blue) and non-pCR (red) according to the log HER2/CEP17 ratio in HR- patients;

**A**


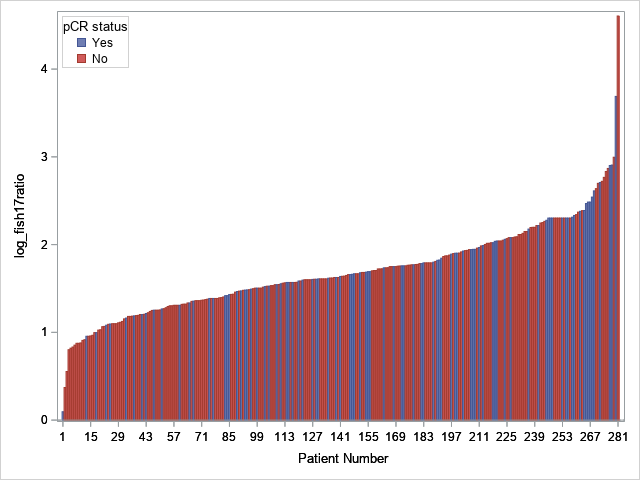


**B**
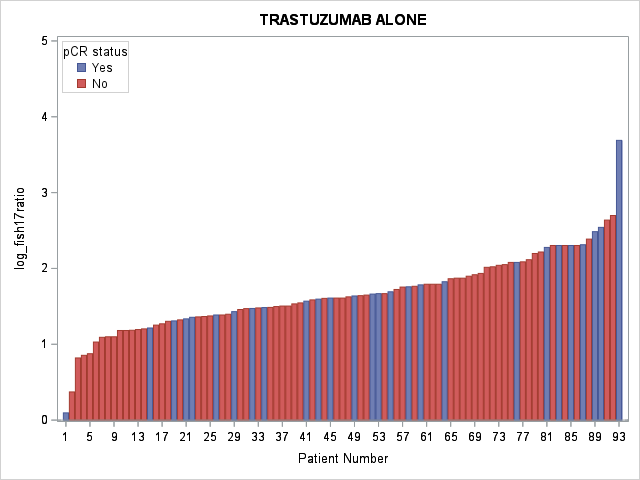


**C**
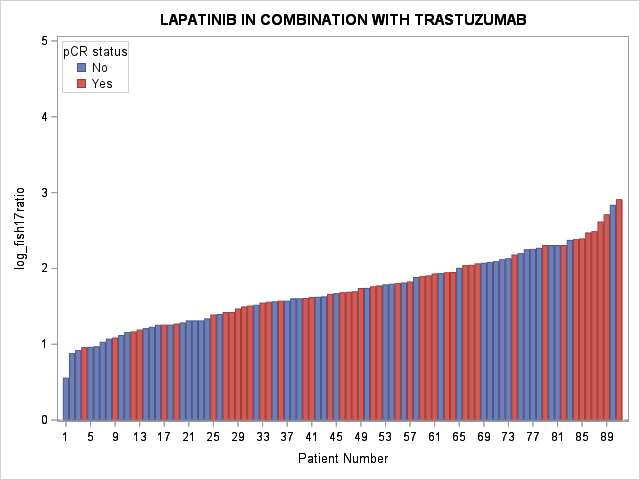


**D**
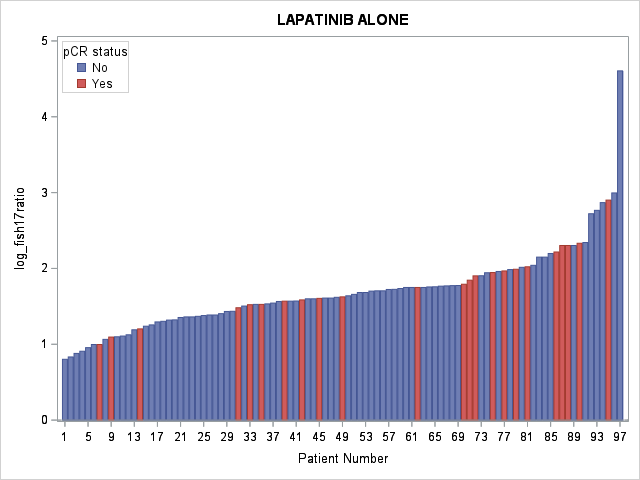


**E**
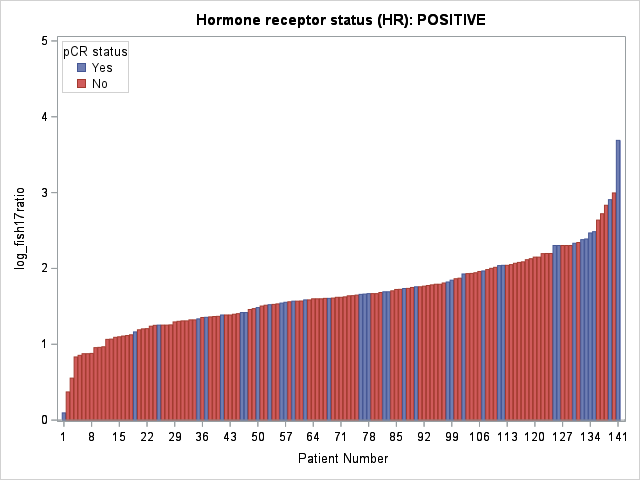


**F**
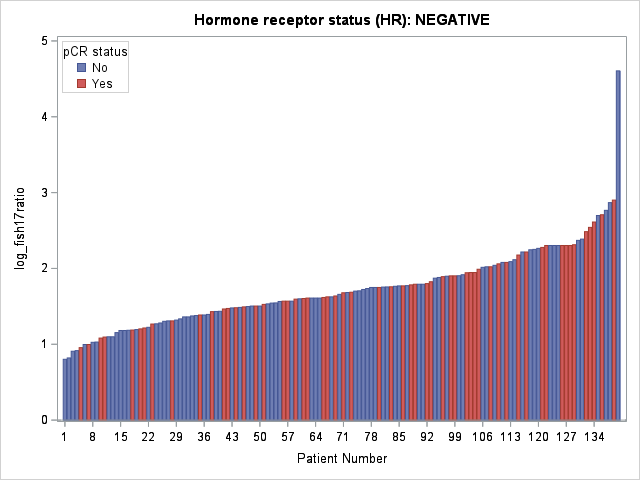


**Figure 3:** Distribution of the logarithmic HER2/FISH ratio according to pCR status in the overall population (A), by treatment arm (B), and by HR status (C)


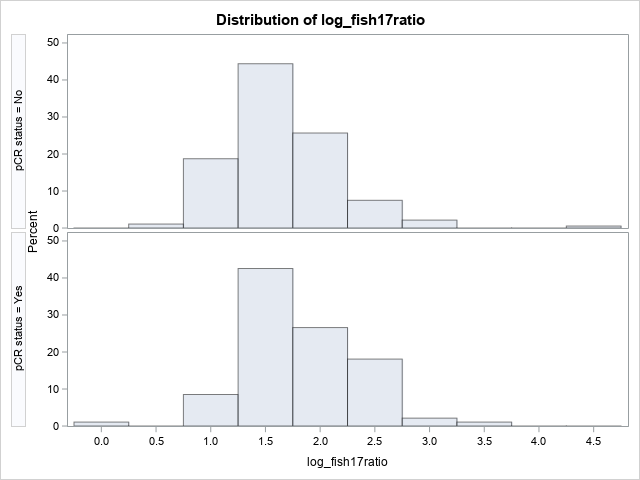
**A**

**B**


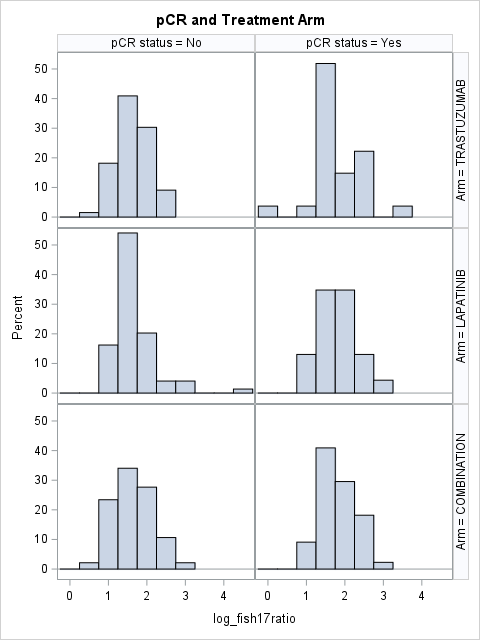


**C**
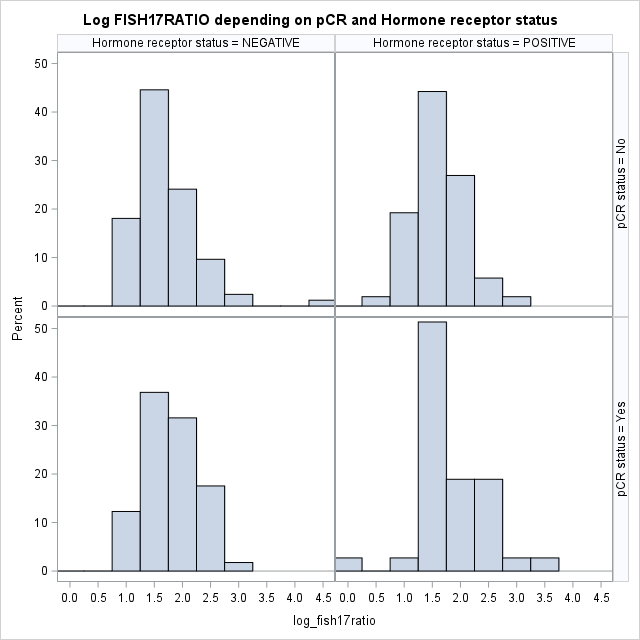


**Figure 4:**  Receiver Operating Curves for the stepwise logistic regression model. Hormone receptor status (brown), treatment arm (green), log HER2/CEP17 ratio (red), combined model comprising HR, treatment arm and log HER2/CEP17 ratio (blue).

**
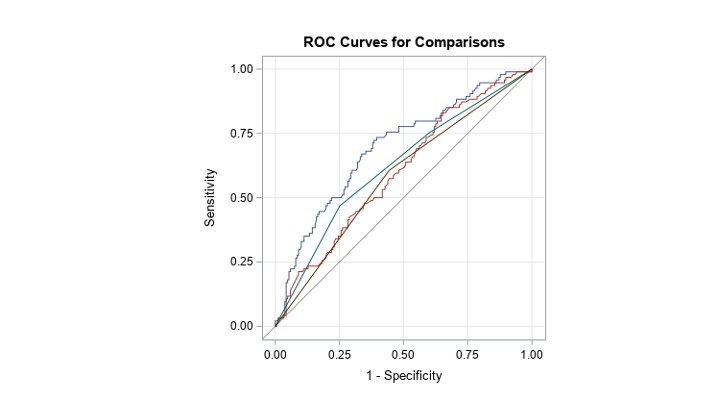
**


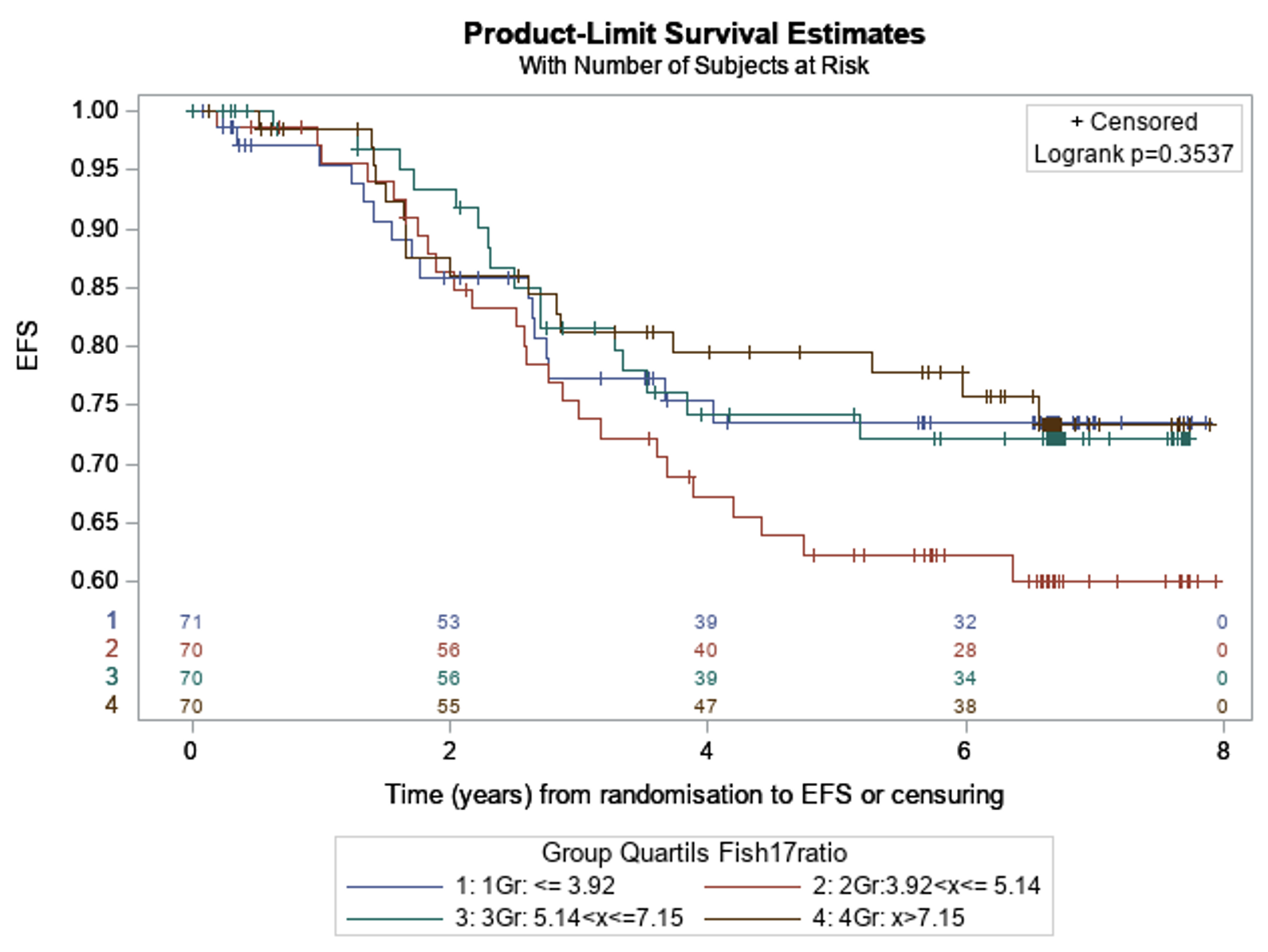
**Figure 5A:** EFS according to the HER2/CEP17 ratio in quartiles (blue: HER2/CEP17 ratio ≤3.92; red: HER2/CEP17 ratio 2.93 - ≤5,14; green: HER2/CEP17 ratio 5.15 - ≤7.15; brown: HER2/CEP17 ratio <7.15).

**Figure 5B:**  OS according to the HER2/CEP17 ratio in quartiles (blue: HER2/CEP17 ratio ≤3.92; red: HER2/CEP17 ratio 3.93 – ≤5.14; green: HER2/CEP17
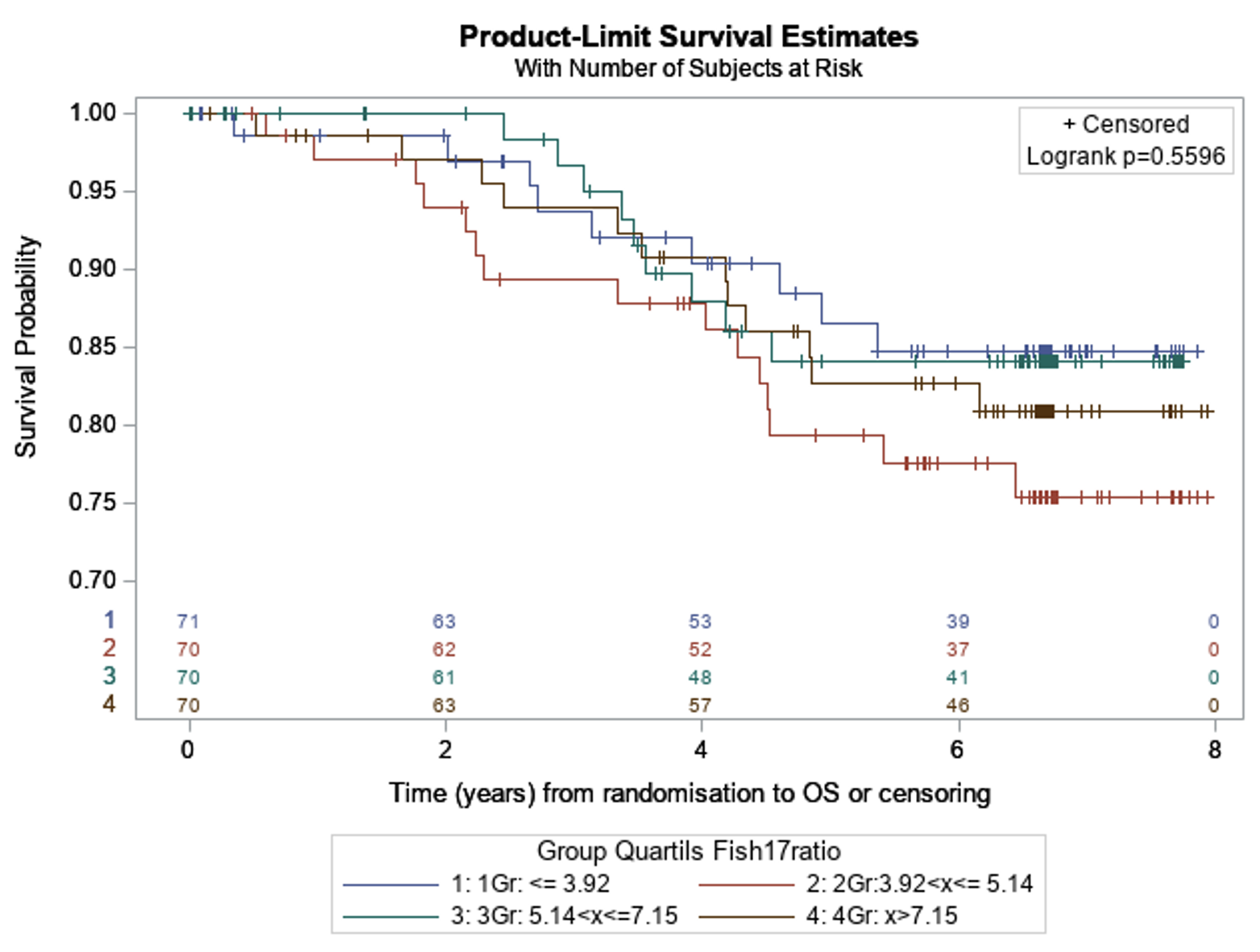
ratio 5.15 - ≤7.15; brown: HER2/CEP17 ratio <7.15).

**ACKNOWLEDGMENTS**

We want to acknowledge all parties involved in the NeoALLTO trial as source of the study data used in the present research project: the Breast International Group (BIG) as study sponsor, the respective Steering Committee, as well as all participating investigators and patients. The NeoALLTO trial was a BIG and SOLTI led trial and financially supported by GlaxoSmithKline until January 2015 and Novartis Pharma AG as of January 2015. The funders had no role in study design or conduction, data collection nor on analysis or interpretation of the present findings.

**REFERENCES**

1. Figueroa-Magalhães MC, Jelovac D, Connolly R, Wolff AC. Treatment of HER2-positive breast cancer. Breast. 2014 Apr;23(2):128-136 [↑](#endnote-ref-2)
2. Oh DY, Bang YJ. HER2-targeted therapies - a role beyond breast cancer. Nat Rev Clin Oncol. 2020 Jan;17(1):33-48. [↑](#endnote-ref-3)
3. Loibl S, Gianni L. HER2-positive breast cancer. Lancet. 2017 Jun 17;389(10087):2415-2429. [↑](#endnote-ref-4)
4. Madden R, Kosari S, Peterson GM, Bagheri N, Thomas J. Lapatinib plus capecitabine in patients with HER2-positive metastatic breast cancer: A systematic review. Int J Clin Pharmacol Ther. 2018 Feb;56(2):72-80. [↑](#endnote-ref-5)
5. Jones J, Takeda A, Picot J, von Keyserlingk C, Clegg A. Lapatinib for the treatment of HER2-overexpressing breast cancer. Health Technol Assess. 2009 Oct;13 Suppl 3:1-6. [↑](#endnote-ref-6)
6. Baselga J, Bradbury I, Eidtmann H, Di Cosimo S, de Azambuja E, Aura C, Gómez H, Dinh P, Fauria K, Van Dooren V, Aktan G, Goldhirsch A, Chang TW, Horváth Z, Coccia-Portugal M, Domont J, Tseng LM, Kunz G, Sohn JH, Semiglazov V, Lerzo G, Palacova M, Probachai V, Pusztai L, Untch M, Gelber RD, Piccart-Gebhart M; NeoALTTO Study Team. Lapatinib with trastuzumab for HER2-positive early breast cancer (NeoALTTO): a randomised, open-label, multicentre, phase 3 trial. Lancet. 2012 Feb 18;379(9816):633-40 [↑](#endnote-ref-7)
7. Nuciforo P, Townend J, Saura C, de Azumbaja E, Hilbers F, Manukyants A, Werutsky G, Bliss J, Moebus V, Colleoni M, Moreno Aspitia A, Di Cosimo S, Van dooren V, Kroep J, Ferro A, Cameron D, Gelber R, Piccart-Gebhart M, Huober J. Nine-year survival outcome of neoadjuvant lapatinib with trastuzumab for HER2-positive breast cancer (NeoALTTO, BIG 1-06): final analysis of a multicentre, open-label, phase 3 randomised clinical trial. European Journal of Cancer. 2020

   Volume 138, Supplement 1, October 2020, S15-S16 [↑](#endnote-ref-8)
8. Fuchs EM1, Köstler WJ, Horvat R, Hudelist G, Kubista E, Attems J, Zielinski CC, Singer CF. High-level ERBB2 gene amplification is associated with a particularly short time-to-metastasis, but results in a high rate of complete response once trastuzumab-based therapy is offered in the metastatic setting. Int J Cancer. 2014 Jul 1;135(1):224-31 [↑](#endnote-ref-9)
9. Singer CF, Tan YY, Fitzal F, Steger GG, Egle D, Reiner A, Rudas M, Moinfar F, Gruber C, Petru E, Bartsch R, Tendl KA, Fuchs D, Seifert M, Exner R, Balic M, Bago-Horvath Z, Filipits M, Gnant M; Austrian Breast and Colorectal Cancer Study Group. Pathological Complete Response to Neoadjuvant Trastuzumab Is Dependent on HER2/CEP17 Ratio in HER2-Amplified Early Breast Cancer. Clin Cancer Res. 2017 Jul 15;23(14):3676-3683 [↑](#endnote-ref-10)
10. Modi S, Park H, Murthy RK, Iwata H, Tamura K, Tsurutani J, Moreno-Aspitia A, Doi T, Sagara Y, Redfern C, Krop IE, Lee C, Fujisaki Y, Sugihara M, Zhang L, Shahidi J, Takahashi S. Antitumor Activity and Safety of Trastuzumab Deruxtecan in Patients With HER2-Low-Expressing Advanced Breast Cancer: Results From a Phase Ib Study. J Clin Oncol. 2020 Jun 10;38(17):1887-1896. [↑](#endnote-ref-11)
11. Bartsch R. Trastuzumab-deruxtecan: an investigational agent for the treatment of HER2-positive breast cancer. Expert Opin Investig Drugs. 2020 Sep;29(9):901-910 [↑](#endnote-ref-12)
12. Carey LA, Berry DA, Cirrincione CT, Barry WT, Pitcher BN, Harris LN, Ollila DW, Krop IE, Henry NL, Weckstein DJ, Anders CK, Singh B, Hoadley KA, Iglesia M, Cheang MC, Perou CM, Winer EP, Hudis CA. Molecular Heterogeneity and Response to Neoadjuvant Human Epidermal Growth Factor Receptor 2 Targeting in CALGB 40601, a Randomized Phase III Trial of Paclitaxel Plus Trastuzumab With or Without Lapatinib. J Clin Oncol. 2016 Feb 20;34(6):542-9. [↑](#endnote-ref-13)
13. Modi S, Jacot W, Yamashita T, Sohn J, Vidal M, Tokunaga E, Tsurutani J, Ueno NT, Prat A, Chae YS, Lee KS, Niikura N, Park YH, Xu B, Wang X, Gil-Gil M, Li W, Pierga JY, Im SA, Moore HCF, Rugo HS, Yerushalmi R, Zagouri F, Gombos A, Kim SB, Liu Q, Luo T, Saura C, Schmid P, Sun T, Gambhire D, Yung L, Wang Y, Singh J, Vitazka P, Meinhardt G, Harbeck N, Cameron DA; DESTINY-Breast04 Trial Investigators. Trastuzumab Deruxtecan in Previously Treated HER2-Low Advanced Breast Cancer. N Engl J Med. 2022 Jul 7;387(1):9-20. [↑](#endnote-ref-14)
14. Wolff AC, Hammond MEH, Allison KH, Harvey BE, Mangu PB, Bartlett JMS, Bilous M, Ellis IO, Fitzgibbons P, Hanna W, Jenkins RB, Press MF, Spears PA, Vance GH, Viale G, McShane LM, Dowsett M. Human Epidermal Growth Factor Receptor 2 Testing in Breast Cancer: American Society of Clinical Oncology/College of American Pathologists Clinical Practice Guideline Focused Update. J Clin Oncol. 2018 Jul 10;36(20):2105-2122. [↑](#endnote-ref-15)
15. Early Breast Cancer Trialists’ Collaborative group (EBCTCG). Trastuzumab for early-stage, HER2-positive breast cancer: a meta-analysis of 13 864 women in seven randomised trials Lancet Oncol. 2021 Aug;22(8):1139-1150. [↑](#endnote-ref-16)
16. Denkert C, Lambertini C, Fasching PA, Pogue-Geile KL, Mano MS, Untch M, Wolmark N, Huang CS, Loibl S, Mamounas EP, Geyer CE, Lucas PC, Boulet T, Song C, Lewis GD, Nowicka M, de Haas S, Basik M. Biomarker Data from the Phase III KATHERINE Study of Adjuvant T-DM1 versus Trastuzumab for Residual Invasive Disease after Neoadjuvant Therapy for HER2-Positive Breast Cancer. Clin Cancer Res. 2023 Apr 14;29(8):1569-1581. [↑](#endnote-ref-17)
17. Mogica JP, Tang H, Liang Y, Zhong M, Hui P, Harigopal M, Krishnamurti U, Fischbach NA, Zhan H. Prognostic impact of reduced HER2 protein expression in post-neoadjuvant therapy resection specimens: A single institution experience and review of the literature. Breast. 2023 Dec;72:103586 [↑](#endnote-ref-18)
18. Tanioka M, Fan C, Parker JS, Hoadley KA, Hu Z, Li Y, Hyslop TM, Pitcher BN, Soloway MG, Spears PA, Henry LN, Tolaney S, Dang CT, Krop IE, Harris LN, Berry DA, Mardis ER, Winer EP, Hudis CA, Carey LA, Perou CM Integrated Analysis of RNA and DNA from the Phase III Trial CALGB 40601Identifies Predictors of Response to Trastuzumab-Based Neoadjuvant Chemotherapy in HER2-Positive Breast Cancer. Clin Cancer Res. 2018 Nov 1;24(21):5292-5304. [↑](#endnote-ref-19)
19. Fernandez-Martinez A, Krop IE, Hillman DW, Polley MY, Parker JS, Huebner L, Hoadley KA, Shepherd J, Tolaney S, Henry NL, Dang C, Harris L, Berry D, Hahn O, Hudis C, Winer E, Partridge A, Perou CM, Carey LA. Survival, Pathologic Response, and Genomics in CALGB 40601 (Alliance), a Neoadjuvant Phase III Trial of Paclitaxel-Trastuzumab With or Without Lapatinib in HER2-Positive Breast Cancer. J Clin Oncol. 2020 Dec 10;38(35):4184-4193. [↑](#endnote-ref-20)
20. Wolff AC, Hammond ME, Schwartz JN, et al. for the American Society of Clinical Oncology, and the College of American Pathologists. American Society of Clinical Oncology/College of American Pathologists guideline recommendations for human epidermal growth factor receptor 2 testing in breast cancer. J Clin Oncol. 2007;25:118–45 [↑](#endnote-ref-21)
21. Cortazar P, Zhang L, Untch M, Mehta K, Costantino JP, Wolmark N, Bonnefoi H, Cameron D, Gianni L, Valagussa P, Swain SM, Prowell T, Loibl S, Wickerham DL, Bogaerts J, Baselga J, Perou C, Blumenthal G, Blohmer J, Mamounas EP, Bergh J, Semiglazov V, Justice R, Eidtmann H, Paik S, Piccart M, Sridhara R, Fasching PA, Slaets L, Tang S, Gerber B, Geyer CE Jr, Pazdur R, Ditsch N, Rastogi P, Eiermann W, von Minckwitz G. Pathological complete response and long-term clinical benefit in breast cancer: the CTNeoBC pooled analysis. Lancet. 2014 Jul 12;384(9938):164-72 [↑](#endnote-ref-22)
22. ? Wolff AC, Hammond ME, Hicks DG, Dowsett M, McShane LM, Allison KH, Allred DC, Bartlett JM, Bilous M, Fitzgibbons P, Hanna W, Jenkins RB, Mangu PB, Paik S, Perez EA, Press MF, Spears PA, Vance GH, Viale G, Hayes DF; American Society of Clinical Oncology; College of American Pathologists. Recommendations for human epidermal growth factor receptor 2 testing in breast cancer: American Society of Clinical Oncology/College of American Pathologists clinical practice guideline update. J Clin Oncol. 2013 Nov 1;31(31):3997-4013.

    ^23^ Fumagalli D, Venet D Ignatiadis M, Azim HA, Maetens M, Rothé F, Salgado R,

    Bradbury I, Pusztai L, Harbeck N Gomez H, Chang T Coccia-Portugal MA, Di Cosimo S, de Azambuja E, de la Peña L, Nuciforo P, Brase JC, Huober J, Baselga J. RNA Sequencing to Predict Response to Neoadjuvant Anti-HER2 Therapy: A Secondary Analysis of the NeoALTTO Randomized Clinical Trial. JAMA Oncol . 2017 February 01; 3(2): 227–234

    ^24^ DeMichele A, Yee D, Berry DA, Albain KS, Benz CC, Boughey J, Buxton M, Chia SK, Chien AJ, Chui SY, Clark A, Edmiston K, Elias AD, Forero-Torres A, Haddad TC, Haley B, Haluska P, Hylton NM, Isaacs C, Kaplan H, Korde L, Leyland-Jones B, Liu MC, Melisko M, Minton SE, Moulder SL, Nanda R, Olopade OI, Paoloni M, Park JW, Parker BA, Perlmutter J, Petricoin EF, Rugo H, Symmans F, Tripathy D, van't Veer LJ, Viscusi RK, Wallace A, Wolf D, Yau C, Esserman LJ. The Neoadjuvant Model Is Still the Future for Drug Development in Breast Cancer Clin Cancer Res. 2015 July 1; 21(13): 2911–2915 [↑](#endnote-ref-23)
